# Institutional Needs

**Published:** 1915-03-06

**Authors:** 


### Institutional Needs.

### "VARIOL."

The need for cleansing preparations in the emergency hospitals which have sprung up since the war has awakened in the minds of a vast body of voluntary hospital workers now enrolled under the Red Cross a sense of the importance of a disinfecting fluid which can be applied to as many different hospital objects as possible. "Variol," which makes no lather like soap and soda, may be used to soften water and cleanse almost any object in hospital. Manufactured by Markham, Sons and Co., Ltd., Siddals Mills, Derby, "Variol" is designed to wash and sweeten ward floors, windows, paintwork, tiles, kitchen tables, sinks and closets, bedpans, ward furniture, from which grease, dirt, and impurities are required to be removed. A powerful cleansing fluid to be used in various degrees of dilution, "Variol" claims attention and trial from all hospital workers, and those especially, perhaps, who are engaged in the difficult but necessary task of preparing buildings, originally designed for very remote purposes, for the use of sick and wounded men. Indeed, it is useful not only for adapted hospitals, but for the men's quarters which have been improvised in buildings, huts, or other structures all over the country. "Variol" as a cleanser of dirt is an institutional preparation which may be used wherever bodies of persons, sick or well, are living a communal life, and therefore for whose welfare sanitation, clean quarters, kitchens, lavatories, dormitories, and wards are absolutely essential.

### HUMPHREYS' PORTABLE BUILDINGS.

As is shown by our notice of the portable hospital now under construction at Liverpool, there was never greater demand for temporary buildings than at the present time, and never greater interest in them. Humphreys, Limited, Knightsbridge, S.W., manufacture and supply seven types of structure, and their catalogue is well worth close study before a decision to embark on any extension or to erect any temporary buildings is made. The first or Standard type has walls and roofs of galvanised corrugated iron, with lining of varnished match-boarding. Italian pattern sheeting, as in type number two, gives a more pleasing appearance to these roofs. In type three the walls are overlaid with timber bands, which can be used so as to give an old-fashioned effect. Walls of corrugated iron, roofs of asbestos cement, lozenge shape tiles, red, white, or grey in colour, are employed in type four. In type five the walls are of asbestos cement fireproof sheeting, the roofs of asbestos cement, with lozenge tiles. In type six a lining of asbestos cement fireproof sheeting replaces the match-boarding. In type seven the walls are of roughcast plaster, with tile roofs. These temporary buildings are capable of prolonged wear and hard work, and the firm's open-air shelters, sleeping chalets, sanatoria buildings, accident wards, and hospitals, isolation or otherwise, are remarkable examples of economy and efficiency in buildings of this type. Humphreys' catalogue is a useful reminder of the varied purposes served by these structures, and should be consulted by all desiring to meet local needs without embarking on unduly expensive extension schemes.

